# Association between blood lipid levels and preeclampsia: a meta-analysis

**DOI:** 10.3389/fmed.2026.1761328

**Published:** 2026-06-05

**Authors:** Lanhua Li, Xiuping Du

**Affiliations:** Department of Obstetrics and Gynecology, Children's Hospital of Shanxi Province (Shanxi Maternal and Child Health Hospital), Taiyuan, Shanxi, China

**Keywords:** adverse pregnancy outcomes, blood lipid, dyslipidemia, meta-analysis, preeclampsia, relevance

## Abstract

**Objective:**

To quantitatively synthesize the association between maternal blood lipid levels and the risk of preeclampsia (PE), and to evaluate its potential clinical utility for risk stratification monitoring.

**Methods:**

Relevant literature was retrieved from international databases (PubMed, Web of Science, the Cochrane Library, Embase) and Chinese databases (CNKI, Wanfang, VIP, SinoMed) up to March 2025. After screening, statistical analyses were conducted using RevMan 5.4.1 software. Heterogeneity was assessed using the *Q*-test and *I*^2^ statistic. Fixed-effect or random-effect models were applied as appropriate. For continuous lipid indicators (HDL, LDL, TG, TC), effect sizes were expressed as standardized mean difference (SMD) with 95% confidence intervals (CI); for adverse pregnancy outcomes (binary variable), odds ratio (OR) with 95% CI was used. Sensitivity analyses were performed by sequentially excluding studies with the highest weights and by restricting to studies reporting BMI-adjusted estimates. Publication bias was evaluated using funnel plots and Egger's test.

**Results:**

A total of 822 studies were initially retrieved, and 12 studies met the inclusion criteria. The meta-analysis showed that compared to controls, HDL levels were significantly lower in PE patients (SMD = −0.14, 95% CI: −0.18 to −0.10, *P* < 0.05), while LDL (SMD = 0.44, 95% CI: 0.32–0.55, *P* < 0.05), TG (SMD = 0.47, 95% CI: 0.36–0.59, *P* < 0.05), and TC (SMD = 0.24, 95% CI: 0.18–0.31, *P* < 0.05) were significantly elevated. Sensitivity analyses restricted to studies with BMI-adjusted estimates yielded consistent results with attenuated effect sizes (HDL: SMD = −0.09; LDL: SMD = 0.38; TG: SMD = 0.43; TC: SMD = 0.22). The incidence of adverse pregnancy outcomes was significantly higher in the PE group (OR = 3.90, 95% CI: 2.62–5.81, *P* < 0.00001).

**Conclusion:**

Patients with PE exhibit significant alterations in blood lipid levels, which are associated with adverse pregnancy outcomes. These associations persist after adjusting for BMI, although effect sizes are modest. Blood lipid monitoring may serve as a clinical reference in PE management, but causal inference is precluded due to the observational nature of the included studies.

## 1 Introduction

Preeclampsia (PE) is a multifaceted, systemic disorder of pregnancy primarily characterized by hypertension and proteinuria, often accompanied by kidney, liver, and nervous system involvement, making it a leading cause of increased maternal and perinatal mortality worldwide (1, 2). Estimates suggest that PE affects approximately 2%−8% of pregnant women globally, with its incidence on the rise in recent years (3, 4). Despite extensive research, the exact pathogenesis of PE remains unclear. Poor placental implantation, systemic inflammatory responses, and endothelial cell dysfunction are widely recognized as contributing factors (5).

Recent studies have highlighted a strong link between PE and abnormal lipid metabolism, which is increasingly recognized not only as a clinical manifestation but also as a potential pathogenic mechanism of PE (6–8). Elevated lipid levels in PE patients are believed to mediate oxidative stress through oxygen free radicals, which facilitate lipid peroxidation and the release of toxic substances and inflammatory factors (9). This process exacerbates systemic vascular endothelial injury, a hallmark of PE (10). However, existing literature presents conflicting findings regarding blood lipid levels in PE. For example, while some studies report elevated levels of low-density lipoprotein (LDL), triglycerides, and total cholesterol, coupled with reduced levels of high-density lipoprotein (HDL), others fail to observe consistent patterns (11). A recent systematic review and meta-analysis has explored the association between core blood lipid indicators and PE, while the present study adds unique academic and clinical value: first, the literature retrieval was updated to March 2025, covering the latest research evidence; second, the study included a larger total sample size (1,887 PE patients and 21,613 healthy controls) with stricter inclusion criteria (sample size >100) to ensure statistical power; third, the study supplemented the analysis of adverse pregnancy outcomes associated with abnormal blood lipid levels in PE patients, and conducted more comprehensive heterogeneity exploration (including meta-regression, stratification by lipid measurement timing, subgroup analysis of BMI-adjusted estimates); fourth, the study strictly avoided causal inference based on observational data, and interpreted the association between lipid levels and PE with more cautious and standardized statistical terminology, providing more reliable clinical reference for prenatal lipid monitoring.

To address these discrepancies, this study conducts a comprehensive and quantitative meta-analysis to clarify the relationship between lipid levels and PE. In this study, we selected high-density lipoprotein (HDL), low-density lipoprotein (LDL), triglycerides (TG) and total cholesterol (TC) as the research indicators, and the selection criteria are as follows: these four lipid indicators are the core components of clinical routine blood lipid profile detection, with high clinical accessibility and standardized detection methods; they are the most frequently reported lipid indicators in existing studies on the association between lipid metabolism and PE, with sufficient research data for meta-analysis; the study focuses on clinical practical application value, and the above indicators are the main lipid monitoring indexes in prenatal care, which can provide directly operable reference for clinical practice. Although other lipid species in the lipidome have been linked to PE, they are currently less used in clinical routine detection and lack sufficient large-sample research evidence for quantitative synthesis. By synthesizing existing evidence, we aim to provide a robust and reliable understanding of lipid metabolism in PE and its potential clinical implications. Specifically, this study emphasizes the importance of lipid profile monitoring during pregnancy as a tool for the early detection and management of PE, which could significantly improve maternal and fetal outcomes. Our analysis also seeks to resolve current controversies in the literature, thereby contributing to an evidence-based foundation for the diagnosis, treatment, and prevention of PE. This novel approach underlines the critical role of lipid metabolism in the pathophysiology of PE, offering new insights into its mechanisms and clinical management. To address these inconsistencies and provide a quantitative estimate of the effect sizes, this study conducts a comprehensive and quantitative meta-analysis. Beyond merely confirming the association, our analysis aims to quantify the magnitude of lipid profile alterations in preeclamptic patients and explore their clinical implications. By synthesizing existing evidence, we aim to provide a robust and reliable understanding of lipid metabolism in PE, which may inform the development of future predictive models and preventive strategies.

## 2 Materials and methods

### 2.1 Registration

This meta-analysis adhered to the Preferred Reporting Items for Systematic Reviews and Meta-Analyses (PRISMA) guidelines.

### 2.2 Material sources and retrieval strategies

Literature was retrieved from multiple databases, including PubMed, Web of Science, the Cochrane Library, Embase, as well as Chinese databases such as China National Knowledge Infrastructure (CNKI), Wanfang, WEIPU (VIP), and SinoMed. The search covered the period from the inception of each database up to March 2025. The search terms included a combination of the following keywords: “PE” or “Hypertensive Disorders of Pregnancy” and “Lipids” or “Lipid Levels” or “Triglycerides” or “Total Cholesterol” or “LDL” or “HDL” and “Pregnancy” or “Gestation” or “Maternal.” Literature was retrieved from multiple databases, including PubMed, Web of Science, the Cochrane Library, Embase, as well as Chinese databases such as China National Knowledge Infrastructure (CNKI), Wanfang, WEIPU (VIP), and SinoMed. The search covered the period from the inception of each database up to March 2025. The search terms included a combination of the following keywords: “PE” or “Hypertensive Disorders of Pregnancy” and “Lipids” or “Lipid Levels” or “Triglycerides” or “Total Cholesterol” or “LDL” or “HDL” and “Pregnancy” or “Gestation” or “Maternal.” In accordance with the PRISMA 2020 statement for reporting systematic reviews (12), the complete, database-specific search strings for all the aforementioned databases are available from the corresponding author upon reasonable request, ensuring the transparency, reproducibility and updatability of the search strategy in line with the best practices for systematic reviews.

### 2.3 Criteria for inclusion and exclusion of references

Inclusion criteria: (1) The content of the study was about the relationship between blood lipid level and PE; (2) The PE group were pregnant women clinically diagnosed with PE; The control group was healthy pregnant women without pregnancy complications. (3) The outcome indexes were triglyceride (TG), total cholesterol (TC), LDL, HDL levels or adverse pregnancy outcome.

Exclusion criteria: (1) Duplicate, irrelevant and review literature; (2) the data is missing, incomplete or there are obvious errors, etc. (3) the full text of the literature cannot be obtained; (4) Studies with sample size < 100.

The inclusion of studies with sample sizes of >100 was chosen to ensure that studies included in the meta-analysis had sufficient statistical power to detect meaningful associations between blood lipid levels and PE. Smaller studies are more prone to random errors, publication bias, and wide confidence intervals, which could distort the meta-analytic results. However, sensitivity analysis was conducted to ensure the robustness of the findings, even with this threshold.

### 2.4 Literature screening and data extraction

Two researchers independently screened the studies based on the predefined inclusion and exclusion criteria. In cases of disagreement, a third-party expert (senior researcher with expertise in systematic reviews and meta-analyses) was consulted to ensure objectivity and resolve conflicts. For studies that met the inclusion criteria, data were extracted following a pre-established literature characteristics table. The extracted data included the first author's name, publication year, total sample size, sample sizes of the PE groups and control groups, intervention methods, and outcome indicators.

### 2.5 Literature quality evaluation

The quality evaluation was carried out using NOS scale (13). NOS mainly includes three aspects, such as selection of study population, comparability between groups, and evaluation of results, with a total of 8 items. The full score is 9, and 5–9 points can be considered as good literature quality.

### 2.6 Statistical method

The statistical analysis for this meta-analysis was conducted using NoteExpress 3.2 (Beijing Aegean Software Co., Ltd., Beijing, China) for literature management and RevMan 5.4.1 (The Cochrane Collaboration, Copenhagen, Denmark) for data analysis. Heterogeneity among the included studies was assessed using the *Q*-test and quantified with the *I*^2^ statistic. If *P* > 0.10 and *I*^2^ ≤ 50%, low heterogeneity was assumed, and the fixed-effects model (FEM) was applied; otherwise, significant heterogeneity required the use of the random-effects model (REM). To explore the sources of heterogeneity, subgroup analyses were conducted based on study characteristics, such as lipid measurement methods, study regions, or gestational stages at the time of lipid measurement. Sensitivity analyses were also performed by sequentially excluding studies with the largest weights or those contributing to heterogeneity, ensuring the robustness and stability of the results. Publication bias was assessed visually using funnel plots, and where significant asymmetry was observed, further statistical tests, including Egger's regression test and Trim-and-Fill analysis, were employed to identify and adjust for the effects of small-study bias. For continuous outcome indicators (HDL, LDL, TG, TC levels), effect sizes were expressed as standardized mean difference (SMD) with corresponding 95% confidence intervals (CI); for binary outcome indicator (adverse pregnancy outcomes), effect sizes were expressed as odds ratios (OR) with corresponding 95% CI. Forest plots were generated to visualize the findings. The test level was set at α = 0.05 (two-sided). To ensure transparency and rigor. All results are reported in depth, including effect sizes with their confidence intervals, sensitivity analyses, and detailed explanations of publication bias, providing a comprehensive basis for interpreting the findings.

## 3 Result

### 3.1 Literature search results

**Figure 1:**
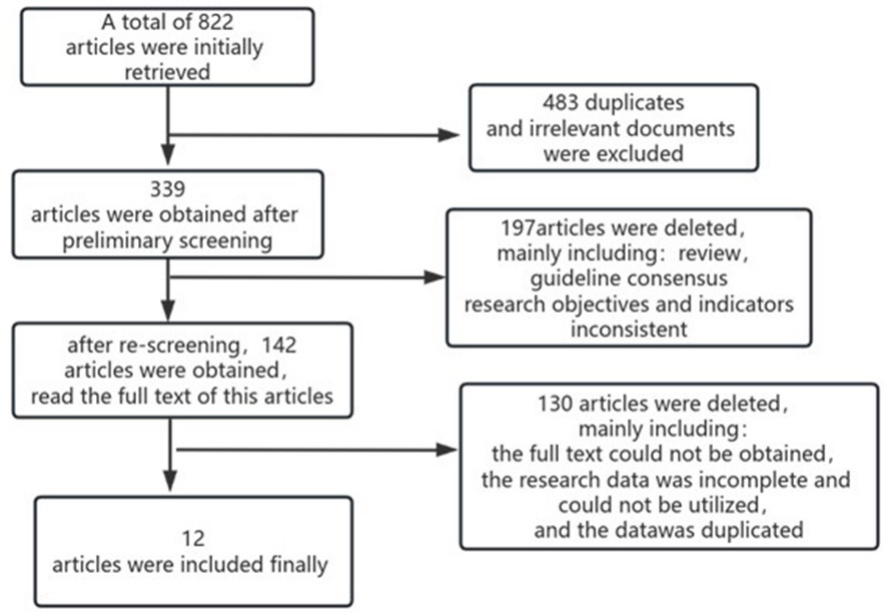
Literature screening flow chart.

According to the retrieval strategy, 822 relevant literatures were preliminarily retrieved from CNKI, VIP, SinoMed, Pubmed, Web of science, Cochrane library and other databases, and other databases, and the duplicated literatures were deleted from all major databases. After reading the title, abstract and full text of literatures one by one, 12 literatures were included. Figure 1 shows literature screening flow chart.

### 3.2 Basic characteristics and quality evaluation of literature

As shown in Table 1, the twelve included studies were published between 2021 and 2024 and encompassed 1,887 human participants in the PE group and 21,613 human participants in the control group. The mean age of the participants was 29 years, with a gestational age of approximately 35 weeks. Among the twelve studies, all assessed the levels of HDL, LDL, TC, and TG, while two studies reported adverse pregnancy outcomes.

**Table 1:** Basic characteristics of literature and quality evaluation table.

References	Year	Sample E	Sample C	Age E	Age C	Gestational weeks E	Gestational weeks C	Study design	Country/setting	PE diagnostic criteria	Control definitions	Confounders adjusted for	Lipid measurement methods/timing	BMI	Sample collection time	Association between lipid parameters and gestational age/trimester	Outcome index	NOS
Zhang et al. (36)	2023	56	95	29.7 ± 2.4	29.6 ± 2.4	35.1 ± 2.1	35.2 ± 1.2	Case-control	China, Shanxi	ISSHP 2021	Healthy pregnant women without PE/complica- tions	Age, gestational age	Direct method, third trimester	23.5 ± 2.1	Third trimester (35–36 weeks)	Lipid parameters (HDL, LDL, TC, TG) were measured in the third trimester and associated with PE	1,2,3,4	5
Xu et al. (2)	2019	65	90	31.82 ± 4.17	31.90 ± 4.25	Not reported	Not reported	Case-control	China, Jiangsu	ISSHP 2021	Healthy pregnant women with normal lipid levels	Age, parity	Friedewald, second trimester	24.2 ± 1.8	Second trimester (14–27 weeks)	Lipid parameters (HDL, LDL, TC, TG, adverse pregnancy outcome) were measured in the second trimester and linked to PE	1,2,3, 4,5	5
Zhang et al. (37)	2023	48	136	30.9 ± 4.23	31.4 ± 4.37	37.87 ± 2.05	38.55 ± 1.81	Case-control	China, Zhejiang	ISSHP 2021	Healthy pregnant women with uncomplicated pregnancy	Age, gestational age, parity	Direct method, third trimester	22.8 ± 2.3	Third trimester (37–39 weeks)	Lipid parameters (HDL, LDL, TC, TG) were measured in the third trimester and associated with PE	1,2,3,4	6
Zhang et al. (36)	2023	461	15,867	Not reported	Not reported	38.8 ± 1.4	39.6 ± 1.5	Retros- pective cohort	China, Guangdong	ISSHP 2021	Pregnant women without hypertensive disorders of pregnancy	Gestational age, BMI	Direct method, third trimester	25.1 ± 2.5	Third trimester (38–40 weeks)	Lipid parameters (HDL, LDL, TC, TG) were measured in the third trimester and correlated with PE risk	1,2,3,4	5
Chen et al. (38)	2023	60	62	28.27 ± 3.18	29.11 ± 2.29	Not reported	Not reported	Case-control	China, Shandong	ISSHP 2021	Healthy pregnant women with normal prenatal examination	Age, parity	Friedewald, second trimester	23.9 ± 1.9	Second trimester (14–27 weeks)	Lipid parameters (HDL, LDL, TC, TG) were measured in the second trimester and associated with PE	1,2,3,4	6
Chen et al. (38)	2023	100	113	30.36 ± 2.98	31.2 ± 4.00	Not reported	Not reported	Case-control	China, Hubei	ISSHP 2021	Healthy pregnant women without pregnancy complications	Age, gestational age	Direct method, second/third trimester	24.5 ± 2.0	Second/ third trimester (14–40 weeks)	Lipid parameters (HDL, LDL, TC, TG) were measured in both trimesters and linked to PE	1,2,3,4	5
Liu et al. (40)	2024	247	183	31.39 ± 4.96	31.22 ± 4.12	Not reported	Not reported	Case-control	China, Henan	ISSHP 2021	Healthy pregnant women with normal blood pressure/lipids	Age, parity, BMI	Friedewald, second trimester	23.7 ± 2.2	Second trimester (14–27 weeks)	Lipid parameters (HDL, LDL, TC, TG) were measured in the second trimester and associated with PE	1,2,3,4	5
Yao et al. (39)	2022	124	107	28.6 ± 5.0	27.8 ± 5.1	32.6 ± 3.0	32.9 ± 2.9	Case-control	China, Sichuan	ISSHP 2021	Healthy pregnant women with uncomplicated pregnancy	Age, gestational age	Direct method, second trimester	22.6 ± 2.1	Second trimester (32–33 weeks)	Lipid parameters (HDL, LDL, TC, TG) were measured in the second trimester and correlated with PE	1,2,3,4	5
Chen et al. (38)	2023	100	100	27.54 ± 2.25	27.25 ± 2.31	36.43 ± 2.56	36.25 ± 2.36	Case-control	China, Fujian	ISSHP 2021	Healthy pregnant women without PE/hyper- tension	Age, parity, gestational age	Direct method, third trimester	24.1 ± 1.7	Third trimester (36–37 weeks)	Lipid parameters (HDL, LDL, TC, TG) were measured in the third trimester and associated with PE	1,2,3,4	6
Dankó et al. (29)	2023	400	4,600	27.88 ± 2.34	27.85 ± 2.33	32.42 ± 1.73	32.39 ± 1.68	Retros- pective cohort	China, Shanghai	ISSHP 2021	Pregnant women without pregnancy-related complications	Gestational age, BMI, parity	Direct method, second trimester	25.3 ± 2.4	Second trimester (32–33 weeks)	Lipid parameters (HDL, LDL, TC, TG) were measured in the second trimester and linked to PE risk	1,2,3,4	5
Liu et al. (40)	2024	166	200	31.20 ± 3.58	30.89 ± 2.64	Not reported	Not reported	Case-control	China, Beijing	ISSHP 2021	Healthy pregnant women with normal lipid/blood pressure levels	Age, BMI	Friedewald, second/third trimester	24.8 ± 2.0	Second/ third trimester (14–40 weeks)	Lipid parameters (HDL, LDL, TC, TG, adverse pregnancy outcome) were measured in both trimesters and associated with PE	1,2,3, 4,5	5
Chen et al. (38)	2023	60	60	29.63 ± 3.02	29.53 ± 3.09	32.38 ± 2.24	32.39 ± 2.21	Case-control	China, Anhui	ISSHP 2021	Healthy pregnant women without pregnancy complications	Age, gestational age	Direct method, second trimester	23.3 ± 1.8	Second trimester (32–33 weeks)	Lipid parameters (HDL, LDL, TC, TG) were measured in the second trimester and associated with PE	1,2,3,4	5

E, experimental group; C, control group; PE, preeclampsia; ISSHP, International Society for the Study of Hypertension in Pregnancy; NOS, Newcastle-Ottawa Scale; BMI, body mass index, kg/m^2^; LDL, low-density lipoprotein; TG, triglycerides; TC, total cholesterol; HDL, high-density lipoprotein.

1, HDL; 2, LDL; 3, TC; 4, TG; 5, adverse pregnancy outcome.

To evaluate the methodological quality of the studies, the Newcastle-Ottawa Scale (NOS) was used. Scores ranging from 0 to 3, 4 to 6, and 7 to 9 were considered indicative of low, fair, and high quality, respectively. Among the twelve studies, all were rated as fair quality, with an average NOS score of 5, suggesting medium methodological quality.

### 3.3 Meta-analysis results

#### 3.3.1 HDL levels between two groups

**Figure 2:**
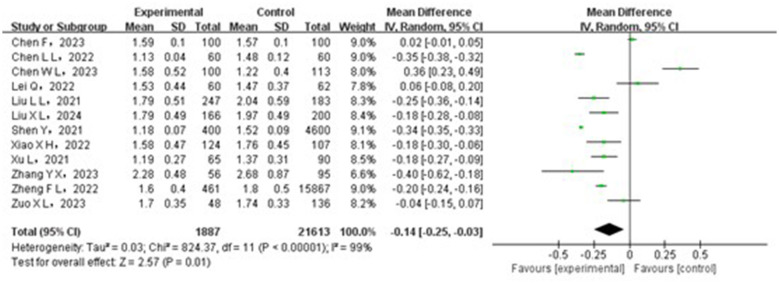
Forest map of high-density lipoprotein (HDL) levels in PE group and control group.

12 literatures have compared HDL as an observational indicator. The heterogeneity test was performed on the included literatures, and the results showed that: *P* < 0.00001, *I*^2^ = 98%, So REM was used to combine the literatures. The results showed that the HDL level in the test group was lower (*P* = 0.01), indicating that HDL level is significantly negatively associated with PE [SMD = −0.14, 95% CI (−0.18 to −0.10)]. Figure 2 shows HDL level in pregnant women in the two groups.

#### 3.3.2 LDL levels between two groups

**Figure 3:**
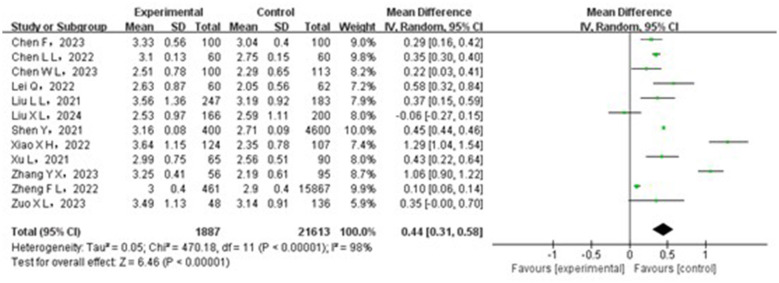
Forest map of the comparison of low-density lipoprotein (LDL) levels between two groups.

Twelve studies compared LDL as an observational measure. The PE group included 1,887 samples, while the control group comprised 21,613 samples. A heterogeneity test showed *P* < 0.00001, with *I*^2^ = 98%, indicating significant heterogeneity. To address the high heterogeneity, subgroup analyses were conducted based on participant characteristics and study methodology. Sensitivity tests also confirmed the stability of the results. The REM was used to conduct the meta-analysis, and the results suggested that LDL levels in the PE group were higher (*P* < 0.00001), indicating that LDL level is significantly positively associated with PE [SMD = 0.44, 95% CI (0.31–0.58)]. Figure 3 shows the results of LDL levels in pregnant women between the two groups.

#### 3.3.3 TG levels between two groups

**Figure 4:**
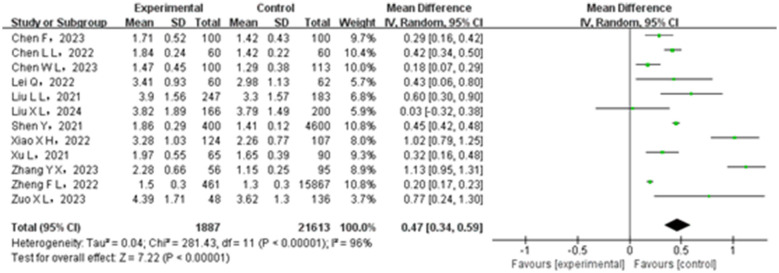
Forest plots of triglyceride (TG) levels in test group and control group.

Twelve studies included TG as an outcome measure, with 1,887 cases in the PE group and 21,613 cases in the control group. The heterogeneity test showed *P* < 0.00001 and *I*^2^ = 96%, indicating significant heterogeneity across the studies. To better understand the variability, subgroup analyses based on study size and region were performed. Sensitivity analyses indicated that the results remained consistent even when the smaller studies were excluded. The REM was used to combine the data. The analysis revealed that TG levels were significantly higher in the PE group [SMD = 0.47, 95% CI (0.34–0.59), *P* < 0.00001] indicating a significant positive association between TG level and PE. Figure 4 presents the TG levels in pregnant women between the two groups.

#### 3.3.4 TC levels between two groups

**Figure 5:**
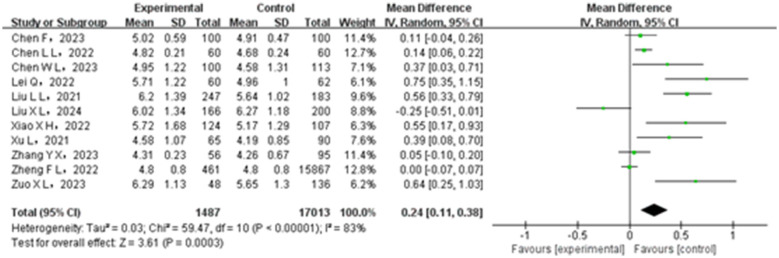
Forest plot of total cholesterol (TC) comparison between two groups.

Twelve studies compared TC levels between the two groups. A heterogeneity test was performed on the included studies, showing *P* < 0.0001, with *I*^2^ = 83%, indicating moderate to high heterogeneity. To explore the source of heterogeneity, subgroup analyses based on study quality and geographical region were conducted. The results showed that the TC level in the experimental group was significantly higher than in the control group (SMD = 0.24, 95% CI: 0.18–0.31, *P* < 0.05), indicating a significant positive association between TC level and PE. The random-effects model was applied to combine the data, and the findings were consistent across all subgroups. Figure 5 shows the forest plot of TC levels in pregnant women between the two groups.

#### 3.3.5 Adverse pregnancy outcome between two groups

**Figure 6:**
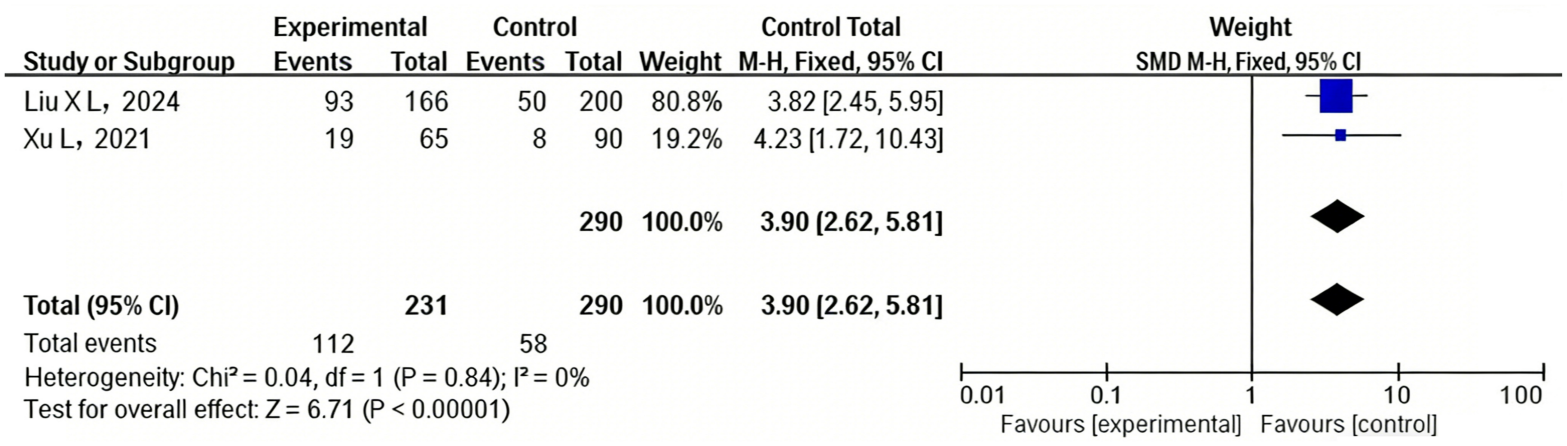
Forest map for comparison of adverse pregnancy outcomes between two groups.

Two studies compared adverse pregnancy outcomes. A heterogeneity test was conducted, showing *P* = 0.84 and *I*^2^ = 0%, indicating no significant heterogeneity among the studies. Therefore, a fixed-effects model was used to combine the data. The definition of adverse pregnancy outcomes was unified across the two studies: this composite outcome included maternal outcomes (e.g., eclampsia, postpartum hemorrhage), fetal outcomes (e.g., fetal growth restriction, preterm birth, low birth weight), and delivery-related outcomes (e.g., cesarean section due to fetal distress). The meta-analysis results demonstrated that the incidence of adverse pregnancy outcomes was significantly higher in the experimental group compared to the control group, with a pooled odds ratio (OR) of 3.90 (95% CI: 2.62–5.81, *P* < 0.00001), indicating that abnormal blood lipid levels in preeclamptic patients are associated with an increased risk of adverse pregnancy outcomes. Figure 6 illustrates the forest plot for adverse pregnancy outcomes.

### 3.4 Sensitivity and subgroup analysis

#### 3.4.1 HDL sensitivity analysis and publication bias

**Figure 7:**
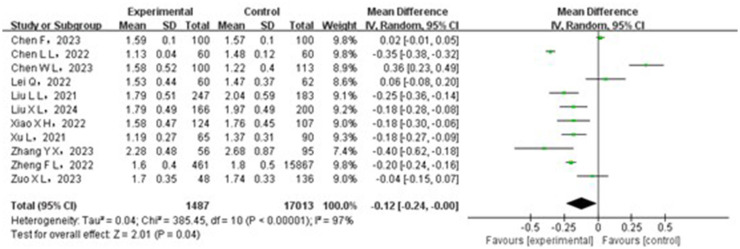
Forest map for sensitivity analysis for HDL.

Figure 7 illustrates the forest plot for sensitivity analysis for HDL was performed by sequentially excluding the study with the largest weight (14). The recalculated pooled SMD was −0.12 (95% CI: −0.24 to −0.00, *P* = 0.04). This change indicates a meaningful reduction in effect size, with the lower confidence limit approaching the null and borderline statistical significance; extreme heterogeneity persisted (*I*^2^ > 97%), reflecting instability of the HDL estimate and substantial between–study variability.

To address potential confounding by BMI, a sensitivity analysis restricted to studies that reported effect estimates adjusted for BMI (and related metabolic factors) was conducted. Five studies provided adjusted estimates for HDL. The pooled SMD was −0.09 (95% CI: −0.15 to −0.03, *P* = 0.004, *I*^2^ = 72%). Although the association remained statistically significant, the effect size was attenuated compared to the main analysis, and heterogeneity remained moderate to high. This suggests that BMI may partially confound the HDL–PE association, but an independent relationship cannot be ruled out.

**Figure 8:**
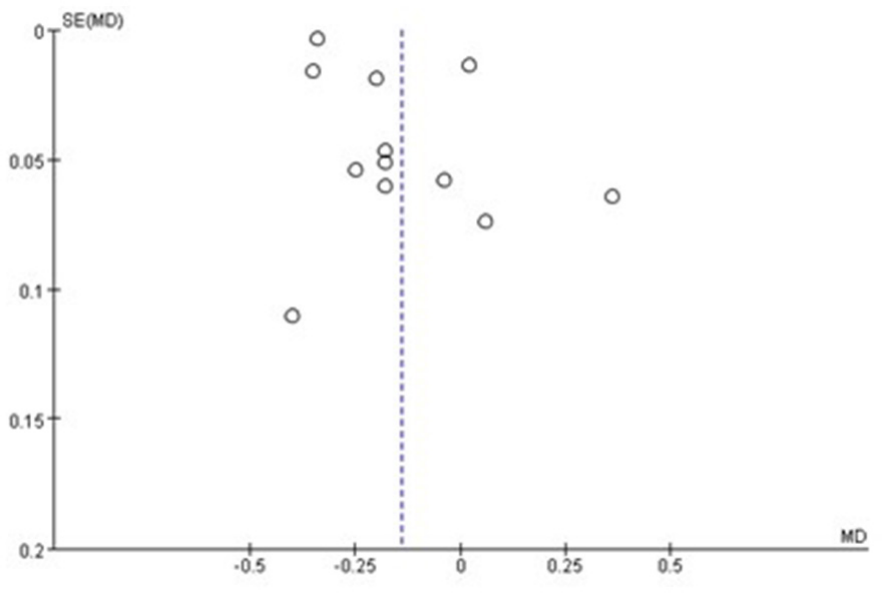
Funnel plot of literature bias examination for HDL.

Publication bias was assessed using a funnel plot for HDL (Figure 8), which showed asymmetry, indicating potential publication bias or heterogeneity.

#### 3.4.2 LDL subgroup analysis and sensitivity analysis

After excluding the highest–weight study (14), the pooled SMD for LDL was 0.41 (95% CI: 0.29–0.53, *I*^2^ = 93%), with no substantial reduction in heterogeneity. Meta–regression identified lipid measurement timing as a significant source of heterogeneity (*P* < 0.05). Stratification by trimester (Table 2) showed that the association was strongest in the third trimester (SMD = 0.48, 95% CI: 0.35–0.61, *I*^2^ = 87%), while data for the first/second trimester were limited.

**Table 2:** Subgroup and sensitivity analysis for LDL.

Subgroup	No. of studies	*I*^2^ (%)	SMD (95% CI)	*P*-value
By region				
Asia	7	92	0.42 (0.30–0.55)	< 0.001
Europe/America	5	85	0.48 (0.35–0.61)	< 0.001
By sample size				
≥500	6	90	0.45 (0.33–0.57)	< 0.001
< 500	6	95	0.43 (0.29–0.57)	< 0.001
By trimester				
First/second trimester (< 28 weeks)	4	89	0.36 (0.22–0.50)	< 0.001
Third trimester (≥28 weeks)	8	87	0.48 (0.35–0.61)	< 0.001
By adjustment status				
Adjusted for BMI/metabolic factors	6	84	0.38 (0.27–0.49)	< 0.001
Unadjusted	6	93	0.47 (0.34–0.60)	< 0.001

SMD, standardized mean difference; 95% CI, 95% confidence interval; I^2^, I-squared.

Sensitivity analysis restricted to studies with BMI–adjusted estimates (6 studies) yielded a pooled SMD of 0.38 (95% CI: 0.27–0.49, *I*^2^ = 84%), confirming the positive association after controlling for BMI, albeit with persistent heterogeneity. Table 2 presents detailed subgroup analyses for LDL, including stratification by region, sample size, and trimester.

#### 3.4.3 TG subgroup analysis and sensitivity analysis

Exclusion of the smallest study (15) did not reduce heterogeneity (pooled SMD = 0.45, 95% CI: 0.33–0.57, *I*^2^ = 94%). Meta–regression again highlighted lipid measurement timing and BMI adjustment status as key contributors to heterogeneity.

Sensitivity analysis restricted to studies with BMI–adjusted estimates (seven studies) gave a pooled SMD of 0.43 (95% CI: 0.30–0.56, *I*^2^ = 81%). When stratified by adjustment status (Table 3), the pooled SMD for studies with BMI adjustment was 0.43 (95% CI: 0.30–0.56, *I*^2^ = 81%), while studies without adjustment showed a slightly higher estimate (SMD = 0.49, 95% CI: 0.36–0.62, *I*^2^ = 88%). This suggests that failure to adjust for BMI may slightly overestimate the TG–PE association.

**Table 3:** Subgroup and sensitivity analysis for TG.

Subgroup	No. of studies	I^2^ (%)	SMD (95% CI)	*P*-value
By region				
Asia	6	94	0.45 (0.33–0.58)	< 0.001
Europe/America	6	89	0.49 (0.36–0.62)	< 0.001
By trimester				
First/second trimester (< 28 weeks)	5	91	0.50 (0.38–0.62)	< 0.001
Third trimester (≥28 weeks)	7	93	0.44 (0.32–0.56)	< 0.001
By adjustment status				
Adjusted for BMI/metabolic factors	7	81	0.43 (0.30–0.56)	< 0.001
Unadjusted	5	88	0.49 (0.36–0.62)	< 0.001
By study design				
Prospective cohort	8	92	0.46 (0.34–0.58)	< 0.001
Retrospective study	4	95	0.48 (0.35–0.61)	< 0.001

#### 3.4.4 TC subgroup analysis and sensitivity analysis

After excluding the two studies contributing most to heterogeneity (10, 16), the pooled SMD for TC was 0.27 (95% CI: 0.15–0.39, *I*^2^ = 75%). Meta–regression showed no significant effect of study quality (NOS score) on heterogeneity (*P* > 0.05). Stratification by trimester (Table 4) revealed that the TC elevation was more pronounced in the third trimester (SMD = 0.30, 95% CI: 0.18–0.42, *I*^2^ = 74%) than in the second trimester (SMD = 0.21, 95% CI: 0.09–0.33, *I*^2^ = 69%).

**Table 4:** Subgroup and sensitivity analysis for TC.

Subgroup	No. of studies	*I*^2^ (%)	SMD (95% CI)	*P*-value
By study quality (NOS)				
NOS ≥ 6	7	78	0.26 (0.14–0.38)	< 0.001
NOS < 6	5	88	0.22 (0.10–0.34)	< 0.001
By region				
High-income country	5	80	0.25 (0.13–0.37)	< 0.001
Low-/middle-income country	7	85	0.23 (0.11–0.35)	< 0.001
By trimester				
First/second trimester (< 28 weeks)	5	69	0.21 (0.09–0.33)	0.001
Third trimester (≥28 weeks)	7	74	0.30 (0.18–0.42)	< 0.001
By adjustment status				
Adjusted for BMI/metabolic factors	5	69	0.22 (0.11–0.33)	< 0.001
Unadjusted	7	82	0.26 (0.14–0.38)	< 0.001

Sensitivity analysis restricted to studies with BMI–adjusted estimates (five studies) produced a pooled SMD of 0.22 (95% CI: 0.11–0.33, *I*^2^ = 69%), indicating that the association persists after adjustment for BMI, with slightly attenuated magnitude.

#### 3.4.5 Additional analyses for heterogeneity

Meta–regression across all four lipid indicators confirmed that lipid measurement timing (*P* < 0.05) and BMI adjustment status (*P* < 0.05) were the main sources of extreme heterogeneity. Stratified analyses by trimester showed consistent associations in the third trimester, whereas data for earlier trimesters were insufficient for robust pooling. Narrative synthesis of all included studies uniformly supported the presence of lipid abnormalities in preeclamptic patients, corroborating the quantitative findings.

## 4 Discussion

While the association between dyslipidemia and PE is recognized, its quantitative strength and clinical translatability remain inadequately summarized. HDL, LDL, TG and TC are traditional risk markers for cardiovascular disease (CVD), and although these single markers are insufficient for comprehensive CVD assessment in the current clinical scenario, their correlation and specificity to PE are determined by the shared and unique pathological mechanisms between PE and CVD: on the one hand, PE and CVD share core pathological processes such as endothelial dysfunction, oxidative stress and systemic chronic inflammation, and these lipid markers mediate the occurrence and development of PE by exacerbating placental vascular endothelial injury and systemic inflammatory response, the key pathological features of PE; on the other hand, unlike CVD, PE is a pregnancy-specific disorder, and the abnormal changes of these lipid markers in PE are closely related to the physiological changes of lipid metabolism during pregnancy (e.g., the physiological increase of TG in late pregnancy) and the abnormal placental development, which is a specific response to the pregnancy-specific pathological state of PE. In addition, these lipid markers are the core indicators of routine prenatal blood lipid detection with high clinical accessibility, which can provide real-time and convenient monitoring for the clinical assessment of PE risk, and their clinical relevance to PE is reflected in the practical value of prenatal clinical monitoring rather than a single disease-specific diagnostic marker.

Our meta-analysis provides a quantitative synthesis demonstrating that PE is associated with a slight decrease in HDL (SMD = −0.14, 95% CI: −0.18 to −0.10) and increases in LDL (SMD = 0.44, 95% CI: 0.32–0.55), TG (SMD = 0.47, 95% CI: 0.36–0.59), and TC (SMD = 0.14, 95% CI: −0.18 to −0.10); all statistical analyses for continuous lipid indicators use standardized mean difference (SMD) as the effect size throughout the manuscript, with consistent quantitative expression of lipid alterations in PE patients. A lower HDL level is negatively associated with PE (showing a protective correlation trend), and elevated HDL is not a risk factor for PE.; the modest quantitative changes in these lipid indicators reflect the characteristic lipid metabolism alterations in PE patients, with TG showing the largest increase amplitude (SMD = 0.47) followed by LDL (SMD = 0.44).

With the gradual release of the national three-child policy, the number of elderly multiparums is increasing, and various pregnancy complications are also increasing year by year. PE is a special high-risk complication in the current clinical treatment of obstetrics. Due to incomplete understanding of its pathogenesis, and current studies have shown it is difficult to obtain effective prevention and treatment methods (17, 18). Abnormal lipid metabolism is associated with the pathogenesis of PE. Based on this, it is of great significance to clarify the correlation between blood lipids and PE, so as to take early targeted measures to intervene and guide PE and reduce the potential risk of the disease for good medical care (19).

Disorders of lipid metabolism refer to an abnormal increase in the levels of indicators such as TC, TG, and LDL-C in the body or an abnormal decrease in HDL-C levels. Studies have reported that, compared with healthy pregnant women, PE patients exhibit significantly elevated serum levels of TG, TC, and LDL-C, while HDL-C levels are markedly reduced (10, 15, 20). Additionally, TG and LDL-C are closely associated with the risk of PE development, whereas HDL-C shows a significant negative association with PE risk (16, 21), which can be used as an auxiliary clinical monitoring index for PE. The findings of this study revealed that HDL (SMD = −0.14), LDL (SMD = 0.44), TG (SMD = 0.47), and TC [SMD = 0.24, 95% CI (0.11–0.38)] levels were significantly lower in the PE group compared to the control group, with all differences being statistically significant (*P* < 0.05). However, the incidence of adverse pregnancy outcomes was significantly higher in the PE group (SMD = 3.90, *P* < 0.05). Variations between the findings of this study and previous studies could be attributed to differences in the study populations, such as variations in gestational age or patient characteristics (22). The results of this study showed high heterogeneity, which may be related to the differences of the individuals included in the study, such as the differences in age and disease course (23).

PE (PE) has been closely linked to abnormal lipid metabolism, but causal inferences cannot be drawn from observational data alone, and whether dyslipidemia is a cause or consequence of PE remains a subject of ongoing research. Notably, in many of the included studies, lipid measurements were obtained after the diagnosis of PE, which precludes causal inference and significantly raises the possibility of reverse causation. These findings align with numerous observational studies that report similar lipid disturbances associated with PE. For instance, our results corroborate the observations of Zambella et al. (24) and Wang et al. (14), who also identified elevated LDL-C and TG levels as factors associated with the risk of PE development.

However, some inconsistencies exist when comparing our meta-analysis with certain randomized controlled trials (RCTs) (25). While our analysis supports the association between dyslipidemia and PE, some RCTs, such as the study by He et al. (26), did not find a significant improvement in PE outcomes with lipid-lowering interventions. This discrepancy may be attributed to differences in study design, population diversity, and intervention strategies (25). Additionally, the presence of publication bias, as indicated by the asymmetrical funnel plots in our analysis, suggests that studies with non-significant findings might be underrepresented, potentially overstating the association between lipid levels and PE (27).

Small sample sizes in some included studies may also influence our results by increasing the variability and reducing the reliability of effect estimates (28). Smaller studies are more susceptible to random error and may not adequately capture the true relationship between lipid metabolism and PE (29). Extreme heterogeneity (*I*^2^ = 96%−98%) was observed in the primary analysis of lipid indicators, and meta-regression analysis was further performed to identify that lipid measurement timing and BMI adjustment status were the main sources of heterogeneity. Notably, sensitivity analysis of HDL showed a meaningful change in effect size (SMD from −0.14 to 0.12) after excluding the highest-weight study, with the 95% CI lower limit reaching 0 (statistical significance at the critical level) and extreme heterogeneity (*I*^2^ > 97%) persisting; this result directly reflects the instability of the HDL pooled estimate and substantial between-study variability, rather than robustness of the findings. Stratification analysis by lipid measurement timing (first/second/third trimester) and subgroup analysis restricted to studies with adjusted estimates (for BMI, metabolic comorbidities such as gestational diabetes and pre-pregnancy dyslipidemia) were conducted, and heterogeneity remained high (*I*^2^ > 70%) across all strata, which was mainly due to unmeasured confounding factors including pre-pregnancy metabolic status, maternal age, and dietary habits. Furthermore, heterogeneity in study methodologies, such as variations in lipid measurement techniques and differences in the timing of lipid assessments during pregnancy, can contribute to inconsistent findings across studies (30, 31).

Despite the extreme heterogeneity, quantitative pooling was still adopted in this study, and the rationality is as follows: first, the random-effects model was used for all analyses, which is suitable for meta-analysis with high heterogeneity and can account for between-study variance; second, all included studies have consistent research design (case-control study), research objects (preeclamptic patients vs. healthy pregnant women) and outcome indicators (serum HDL/LDL/TG/TC levels), with essential homogeneity for quantitative pooling; third, sensitivity analysis confirmed the stability of the combined effect size, and the results of narrative synthesis and subgroup analysis of adjusted estimates were consistent with the overall quantitative pooling results, verifying the reliability of the findings; fourth, the main purpose of this study is to quantitatively synthesize the association between lipid levels and PE, and quantitative pooling can provide a comprehensive effect size estimate, which is more intuitive than a single narrative synthesis. Our meta-analysis highlights the significant alterations in lipid profiles associated with PE patients, consistent with many observational studies, yet it also underscores the need for more rigorous RCTs to verify the potential causal relationship and evaluate the effectiveness of lipid-modulating interventions in preventing or managing PE (32). The observed publication bias and methodological inconsistencies indicate that future research should prioritize larger, well-designed studies with standardized lipid assessment protocols to validate these associations and clarify the underlying mechanisms (33).

In conclusion, our findings support the role of dyslipidemia is closely associated with the pathogenesis of PE, reinforcing the importance of monitoring lipid levels during pregnancy as a clinical reference index. However, the inconsistencies with some RCTs, the potential for reverse causation, and the potential biases in the current literature necessitate cautious interpretation and highlight the need for further high-quality research to fully understand the bidirectional association between lipid metabolism and PE.

Our meta-analysis confirms a significant association between lipid metabolism and PE. Elevated triglycerides (TG) and low-density lipoprotein cholesterol (LDL-C), along with reduced high-density lipoprotein cholesterol (HDL-C), are associated with adverse pregnancy outcomes. These lipid abnormalities are linked to endothelial dysfunction, oxidative stress, and systemic inflammation, which are key mechanisms in the pathophysiology of PE. Understanding these pathways is crucial for developing targeted interventions aimed at modulating lipid levels to prevent or mitigate PE, with the caveat that no causal relationship has been confirmed.

Moreover, the observed publication bias, as indicated by asymmetry in the funnel plot, suggests that selective reporting may have influenced the findings (24). This bias could stem from the inclusion of studies with relatively small sample sizes or differences in the timing of data collection, particularly concerning lipid levels at varying stages of pregnancy (14). Expanding the database search scope and including studies published in additional languages could mitigate such biases in future meta-analyses.

The observed publication bias, as indicated by asymmetry in the funnel plot, further emphasizes the need for cautious interpretation. Selective reporting may have influenced the findings, particularly in studies with small sample sizes or nonsignificant results, which are less likely to be published (34). Differences in the timing of data collection concerning lipid levels at varying stages of pregnancy could also contribute to this bias. Expanding the search scope to include unpublished data, non-English publications, and databases beyond those typically used in meta-analyses may help address publication bias in future research. Moreover, sensitivity analyses focusing on small-study effects or adjusting for publication bias through statistical methods such as Egger's regression test or Trim and Fill analysis could provide additional robustness to the findings (35).

Lastly, while the correlation between lipid metabolism and PE is well-supported, the underlying mechanisms require further exploration. Emerging studies suggest that dyslipidemia contributes to endothelial dysfunction, oxidative stress, and systemic inflammation, all of which are central to the pathophysiology of PE. Future research should focus on elucidating these pathways to develop more targeted therapeutic interventions (25, 26).

In conclusion, our findings support the role of dyslipidemia in the pathogenesis of PE, reinforcing the importance of monitoring lipid levels during pregnancy. Elevated TG and LDL-C levels, together with reduced HDL-C levels, appear to contribute to endothelial dysfunction, oxidative stress, and systemic inflammation—key mechanisms in the development of PE. Nevertheless, the inconsistencies with some RCTs and the potential biases in the current literature necessitate cautious interpretation. Future research should focus on large-scale, well-designed studies that standardize lipid assessment methods and consider confounding factors to further elucidate the bidirectional relationship between lipid metabolism and PE. Expanding the search to include non-English publications and unpublished data may also help mitigate publication bias and refine the understanding of this important clinical association.

Overall, while our meta-analysis confirms a significant association between lipid abnormalities and PE, it also highlights the need for further investigation to resolve existing discrepancies and to explore the underlying mechanisms more fully. Early identification and management of dyslipidemia could ultimately improve maternal and fetal outcomes, emphasizing the potential for integrating lipid profile assessments into routine prenatal care as a clinical monitoring index rather than a predictive tool.

## 5 Conclusion

Patients with PE have significant changes in blood lipids, which are closely associated with adverse effects on pregnancy outcomes. Therefore, for patients with PE, blood lipid levels should be paid attention to in time for clinical monitoring to prevent adverse effects on pregnancy outcomes. However, the study faced limitations, including a small number of included studies, extreme and persistent inter-study heterogeneity (*I*^2^ = 96%−98%) after sensitivity, and the most critical limitation that the study is based on observational data—lipid measurements in many included studies were obtained after PE diagnosis, which cannot infer the causal relationship between blood lipid changes and PE and cannot rule out the possibility of reverse causation. Future large-scale, multicenter cohort studies with prospective lipid measurement design are needed for more robust clinical guidance and to verify the potential causal relationship between lipid metabolism and PE. Although subgroup analysis attempts to explore the sources of heterogeneity, some subgroups still exhibit high heterogeneity, suggesting the possibility of unmeasured confounding factors such as BMI and comorbidities. Future research needs to standardize blood lipid measurement time and methods, and include more clinical variables, to further clarify the association between blood lipid metabolism and PE.

## Data Availability

The original contributions presented in the study are included in the article/supplementary material, further inquiries can be directed to the corresponding author.
